# Crosslinking of floating colloidal monolayers

**DOI:** 10.1007/s00706-017-1997-6

**Published:** 2017-06-20

**Authors:** Steffen Kurzhals, Michael Süss, Jelena Pejovic, Peter D. J. van Oostrum, Erik Reimhult, Ronald Zirbs

**Affiliations:** 1 Department of Nanobiotechnology, University of Natural Resources and Life Sciences Vienna, Muthgasse 11, 1190 Vienna, Austria; 2 CEITEC, Central European Institute of Technology, Brno University of Technology, Purkyňova 123, 612 00 Brno, Czech Republic

**Keywords:** Colloids, Nanostructures, Crystal structure, Electron microscopy, Assembly, Annealing

## Abstract

**Abstract:**

Crosslinked colloidal monolayers are promising as templates, lithographic masks, filtration membranes, or membranes for controlled release rates in drug delivery. We demonstrate assembly of monodisperse micron-sized polystyrene (PS) beads at an air/water interface, which are transformed into crystalline monolayers using addition of surface-active agents. Vapor annealing methods with solvents (toluene and xylene) and crosslinking agents (divinylbenzene) were investigated regarding their ability to crosslink these floating monolayers directly at the interface, generating crosslinked membranes with crystal size up to 44 cm^2^, domain size up to 1.9 mm^2^, and nano-sized pores (100–300 nm). The demonstrated fabrication method emphasizes short fabrication time using a simple setup.

**Graphical abstract:**

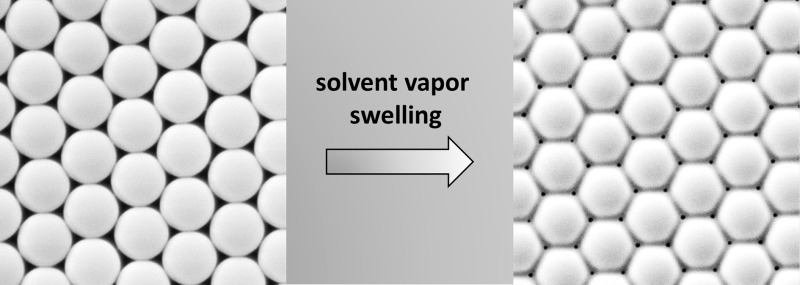

**Electronic supplementary material:**

The online version of this article (doi:10.1007/s00706-017-1997-6) contains supplementary material, which is available to authorized users.

## Introduction

Crystalline, colloidal monolayers have been shown to be highly useful for nanolithography [1–4], structural color painting [5], or as templates for fabrication of porous materials [6–8]. Common assembly methods to prepare these structures include drop casting, spin coating [9–11], evaporation [11] on solid substrates, or self-assembly at air/liquid interfaces with subsequent transfer [1, 12–19]. Polystyrene latex and silica beads are the most commonly used materials, as they can be fabricated at low costs with narrow size distributions. Monodisperse particles are a crucial requirement for highly crystalline order in the assembled masks or templates. Applied for nanolithography, the high order of the mask translates after metal deposition (e.g., cobalt, silver, and gold) in highly ordered arrays of nanodots [1–3], -rods [1], or -rings [1, 4] most useful for electronics, optics, and material science. Analogously, porous materials created as negative replicas of the colloidal templates display a high order in the porous structure [6, 20].

To tune the size of deposited nanostructures or pore sizes, the aperture sizes in the mask can be tuned by the colloid size, commonly in the range of 0.2–2 µm [1, 3, 9, 21], and annealing procedures after the assembly process giving access to sizes in the range of several hundred down to tens of nanometers. Multiple techniques have been used to tweak the aperture size including thermal heating [16], microwave-assisted solvent annealing [1], vapor swelling [2, 16], or chemical modification [3]. PS latex masks could be tuned by microwave-assisted solvent annealing (water/ethanol/acetone) from 200 down to 25 nm [1]. With static [16] and dynamic vapor swelling methods [2] (exposure time: 5–20 min), monolayer films with pore diameters of 150 down to 100 nm and 140 down to 47 nm could be fabricated. Aperture size of silica particle masks was tuned by step-wise coating with TEOS (Stöber process) from the initial pore size of 400 down to 55 nm [3]. Extended exposure with most of the techniques results in disappearance of the apertures and thus to fully closed films. Most of the procedures are not easily reproduced as either a complex setup or equipment is required. Thus, a facile way to prepare crosslinked colloidal monolayers covering large areas with uniform pore size and using a minimum of complex equipment is still highly desired.

Herein, we investigate the static and dynamic vapor swelling of floating monolayers of self-assembled PS beads to fabricate colloidal masks with nano-sized pores with an emphasis on simple setup and short fabrication times.

## Results and discussion

**Fig. 1:**
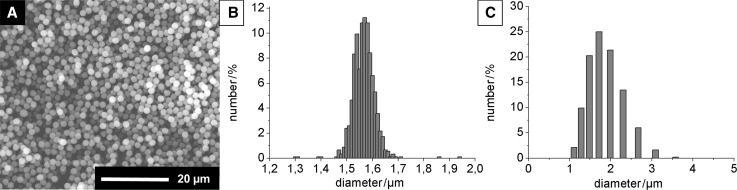
**a** Scanning electron micrograph of PS particles, **b** size distribution by SEM (ImageJ, analysis of ≥900 particles), and **c** size distribution by DLS

The synthesis of polystyrene (PS) particles by precipitation polymerization was done analogously to established methods using polyvinylpyrrolidone (PVP) as surfactant [22, 23]. For self-assembly, devoid of gaps or defects, particles with a narrow size distribution are necessary [24]. Inspection of the particles by scanning electron microscopy (SEM) showed spherical particles. Size determination of ≥900 particles by image analysis gave a narrow size distribution with a mean particle diameter of 1.567 ± 0.043 µm (2.7%), (Fig. 1a, b). Investigation of particle dispersion by DLS gave particles with a number-weighted mean diameter of 1.924 ± 0.352 µm (Fig. 1c), which is slightly larger than the size measured by SEM. Dispersed PS particles are expected to have a larger size compared to dry particles imaged by SEM due to slight swelling and the larger hydrodynamic size measured as solvated PVP chains extend from the particle surface.

### Assembly

**Fig. 2:**
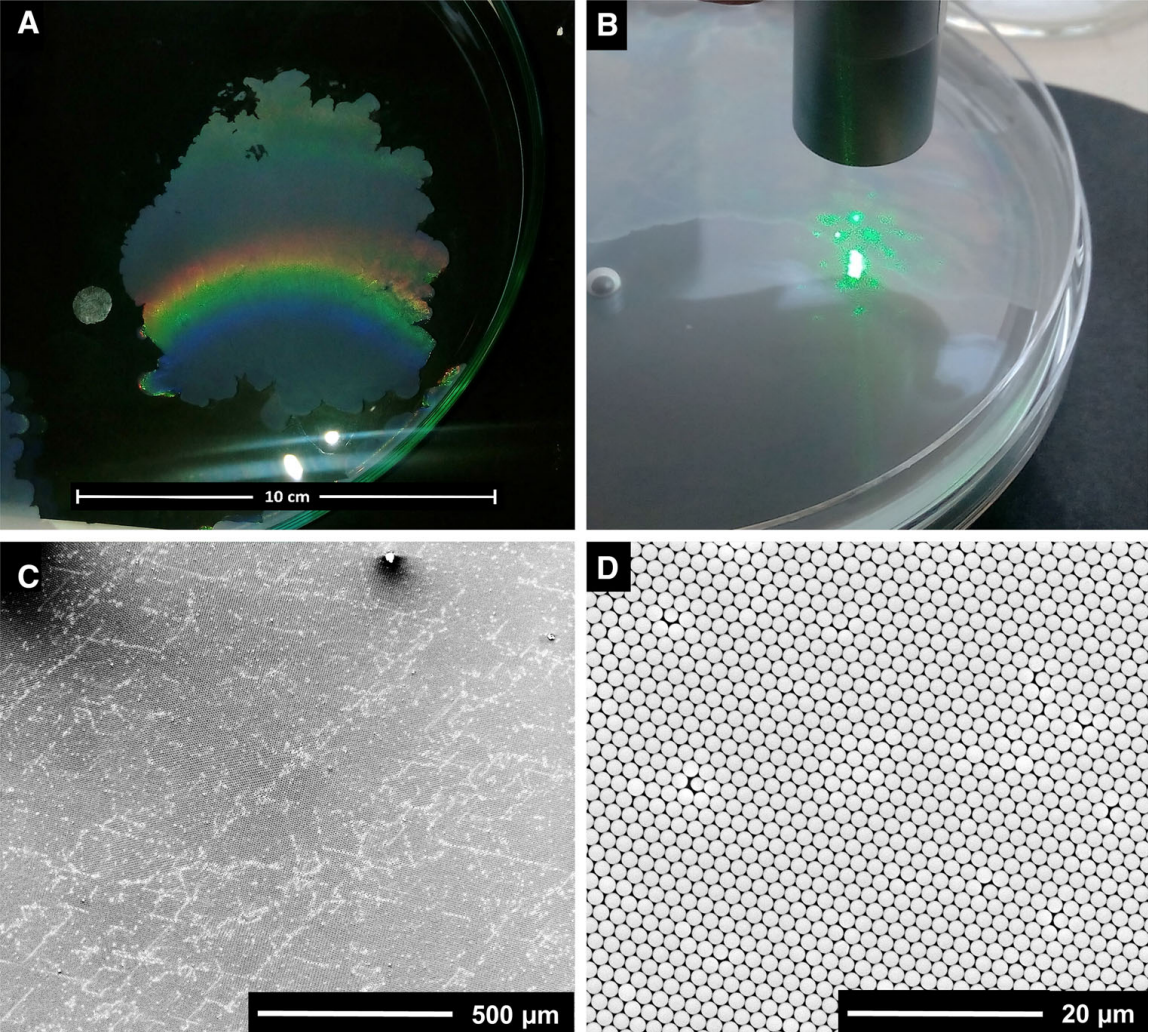
PS particle assembly (≈44 cm^2^) at the air/water interface, gently agitated at 45 rpm for 30 min and compressed by addition of Triton X-100, **a** light refraction of PS monolayer at the air/water interface, **b** laser diffraction pattern showing the high crystallinity of PS monolayer, **c**, **d** scanning electron micrographs of monolayer transferred to silicon substrate, **c** single crystal domain (image size: 1.9 mm^2^)

After successful synthesis of the PS particles, a protocol, previously published by Kandulski [16], was adapted for their assembly (Figs. S1, S2). This method is based on the self-assembly of colloids at the air/water interface. Instead of filling the complete interface with particles, approximately 70% of the available surface area was covered by spreading a dispersion of PS particles in water/ethanol (v/v: 1/1). The sparse monolayer of PS particles at the interface is further treated by a two-step procedure. In the first step, the spread particles are evenly distributed by gentle agitation manually or by shaker at low rpm speeds (45 rpm) for 30 min to create a homogenous distribution. In the second step, the particle monolayer at the interface is condensed and compressed by addition of the surface-active agent Triton X-100. The surfactant competes with the particles for interfacial area and it forces them into a close-packed and highly crystalline assembly (Figs. S1, S2). The assemblies depicted in Fig. 2a and b display Bragg refraction of white light typical for monolayers of monodisperse particles (Fig. 2a) [2, 5].

The generated assemblies were illuminated by a monochromatic laser yielding a hexagonal diffraction pattern as a result of the highly crystalline monolayer (Fig. 2b). However, the entire, floating monolayer is not a single crystal, but composed of single crystal domains, as the orientation of the diffraction pattern changes with moving the laser beam over the assembly.

Using Triton X-100 for film formation had the additional advantage that a pre-wetted silicon wafer (5 × 5 mm) could be dipped into the water phase close to the walls and moved directly beneath the film without disturbing the assembly. By slowly pulling out the silicon wafer, the PS monolayer is transferred to the substrate. The area of the transferred film was only limited by the size of the wafer substrates that were used and could be scaled-up by increasing the size of the substrate. Inspection by SEM of the films transferred to wafers shows homogeneous films with areas up to 11 mm^2^ (Fig. S3A) devoid of cracks. Crystal defects cannot be completely avoided, visible by a low number of vacancies, multilayer defects, and crystal dislocations (Figs. 2c, S3). These defects can originate from compression of the monolayer, particle sedimentation, transfer to the substrate, and/or subsequent drying. Defect densities [16] for Fig. 2c are 12 × 10^4^ particle vacancies cm^−2^, 20 × 10^4^ multilayer particles cm^−2^, and 0.38 m cm^−2^ length of crystal dislocations per area. The transferred films show the desired hcp structure (Fig. 2d), with long-range order of the PS particles and exhibit single crystal domains of up to 1.9 mm^2^ (lower estimate). Aperture sizes in crystal domains are in the range of 385 ± 22 nm. Crystal dislocations, e.g., particles, in square packing will slightly broaden the pore diameter distribution, as their aperture size will be larger even after swelling, compared to analogous pores in hcp configuration. However, as a certain magnification for SEM is necessary to image the pores, pore diameters were analyzed from single crystal domains.

### Crosslinking of particles

After assembly of the PS particles, two different methods were investigated on their ability to create crosslinked films with defined, narrowly distributed pore sizes: static and dynamic vapor swelling. Solvents used for vapor swelling include divinylbenzene, toluene, and xylene (Table 1). In case of divinylbenzene, vapor swelling was followed by UV polymerization for 15 min, thus generating a covalently linked network. For toluene and xylene, annealing relies on physical crosslinking by swelling and subsequent chain entanglement. Crosslinking should allow to transfer films from the interface more easily and to preserve single crystal domain sizes as a result of higher stability of the colloidal monolayer.

**Table 1:** Overview of crosslinking procedures

Procedure	Vapor	Exposure time	Pore diameter/nm	Immediate termination
Before crosslinking	–	–	385 ± 22	–
Static vapor swelling	DVB	5 min + UV^a^	207 ± 19	No
Static vapor swelling	DVB	10 min + UV^a^	184 ± 48	No
Static vapor swelling	Toluene	100 s	104 ± 17	No
Dynamic vapor swelling	Toluene	40 s	304 ± 25	Yes
Dynamic vapor swelling	Xylene	50 s	312 ± 32	Yes
Dynamic vapor swelling	Xylene	60 s	98 ± 12	Yes

^a^After DVB exposure, UV polymerization for 15 min

### Static vapor swelling/UV polymerization for covalent crosslinking

Swelling with DVB vapors was achieved by placing the dish with the floating monolayer in a vapor chamber, depicted in Fig. S4 with a total volume of 942.5 cm^3^, together with a small dish filled with divinylbenzene (0.5 cm^3^) for 5–20 min. In a second step, the dish was placed in an UV chamber and UV polymerization was allowed for 15 min to render the particle monolayer permanently crosslinked. After UV polymerization, the layers were transferred to silica substrates (5 × 5 mm). Originally, a swelling time of 5 min was used. SEM analysis confirmed a reduction in pore size from 385 ± 22 nm down to 207 ± 19 nm (Fig. S5B, D). An increase of the swelling time to 10 min resulted in a further reduction in pore size to 184 ± 48 nm (Fig. S5D). However, apertures obtained under these conditions are slightly irregular (Fig. S5C), evident by the large standard deviation. A closed film was obtained with a swelling time of 20 min.

### Static vapor swelling for physical crosslinking

Analogously to experiments with DVB, static vapor swelling with toluene was performed in a vapor chamber with a total volume of 942.5 cm^3^ (Fig. S4; Table S2). First experiments were performed by placing one to four dishes (diameter: 2.5 cm) filled each with toluene (0.5 cm^3^) in the vapor chamber and exposing the films for 24 h. However, this approach was unsuccessful, as the particle monolayers remained unaltered. Only after placing five dishes with each 0.5 cm^3^ of toluene in the chamber (surface of 24.5 cm^2^ for solvent evaporation) and exposure for 24 h, crosslinked films were observed with a pore diameter of 337 ± 15 nm (Table S2).

**Fig. 3:**
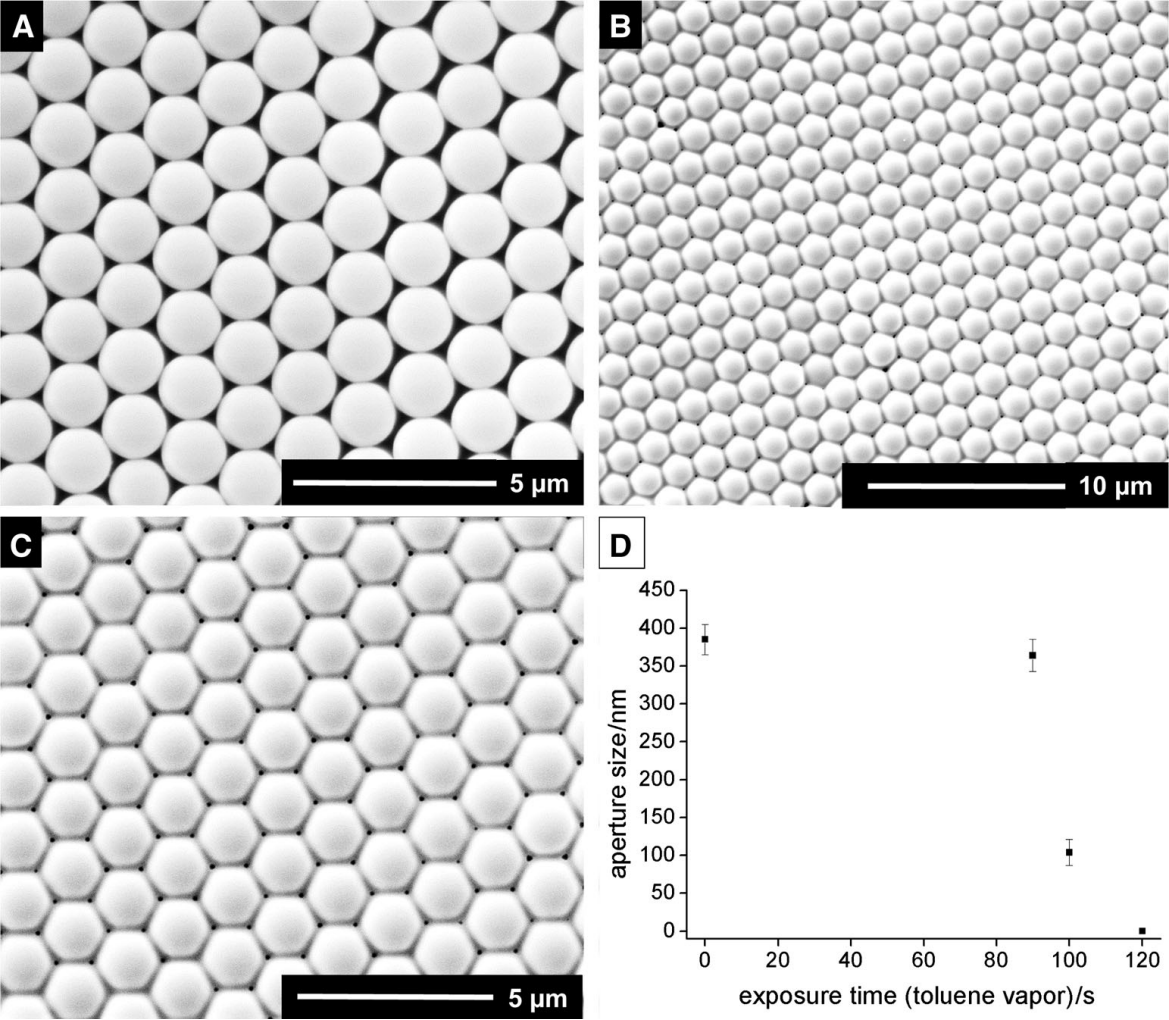
Scanning electron micrographs for PS particle films treated by static vapor swelling, **a** film treated with 14 cm^3^ of toluene in the vapor chamber for 90 s, **b**, **c** film treated with 14 cm^3^ of toluene in the vapor chamber for 100 s, and **d** aperture size vs. exposure time with toluene vapor (aperture size and standard deviation by image analysis with ImageJ for ≥100 apertures)

The process could be significantly accelerated by filling the complete area around the dish with the floating monolayer with toluene (14 cm^3^). In this setup, the particles remain unaltered for 90 s (Fig. 3a). However, already after 100 s, the film is crosslinked with pore diameters of 104 ± 17 nm as determined by SEM (Figs. 3c, S6). The defect density [16] increased going from assembled to crosslinked film from 12 × 10^4^ to 54 × 10^4^ particle vacancies cm^−2^ and from 0.38 to 1 m cm^−2^ for the length of crystal dislocations per area. However, no multilayer defects could be observed. Longer exposure (120 s) leads to fully closed films (Fig. S7). The use of a magnetic stir bar for accelerating the evaporation process on the same time scale even leads to deformed particles.

### Dynamic vapor swelling for physical crosslinking

**Fig. 4:**
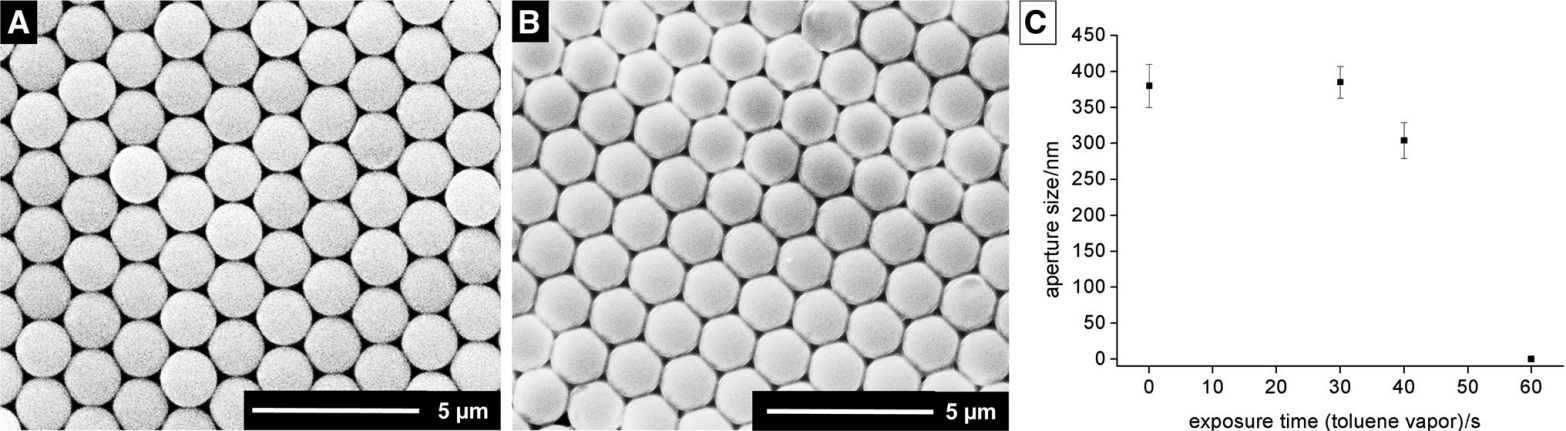
Scanning electron micrographs of PS particle films treated with dynamic vapor swelling (toluene) for **a** 30 s, **b** 40 s, and **c** aperture size vs. exposure time with toluene vapor (aperture size and standard deviation by image analysis with ImageJ for ≥100 apertures)

The experimental setup for the dynamic vapor swelling is given in Fig. S8. The setup consists of a washing bottle, working as solvent reservoir that is connected to the vapor chamber, which is further connected to a vacuum pump. Removal of residual solvent vapors in the chamber by vacuum occurs in approximately ≤0.2 s by simply closing off the vapor chamber. Thus, accurate control over the exposure time is given. The crosslinking process was monitored with time (Fig. 4; Table S3). While exposure for 30 s with toluene vapor had no effect on the particle film (Fig. 4a), crosslinking was observed after exposure for 40 s with pore sizes of 304 ± 25 nm (Fig. 4b, c). A fully closed film was obtained after 60 s.

**Fig. 5:**
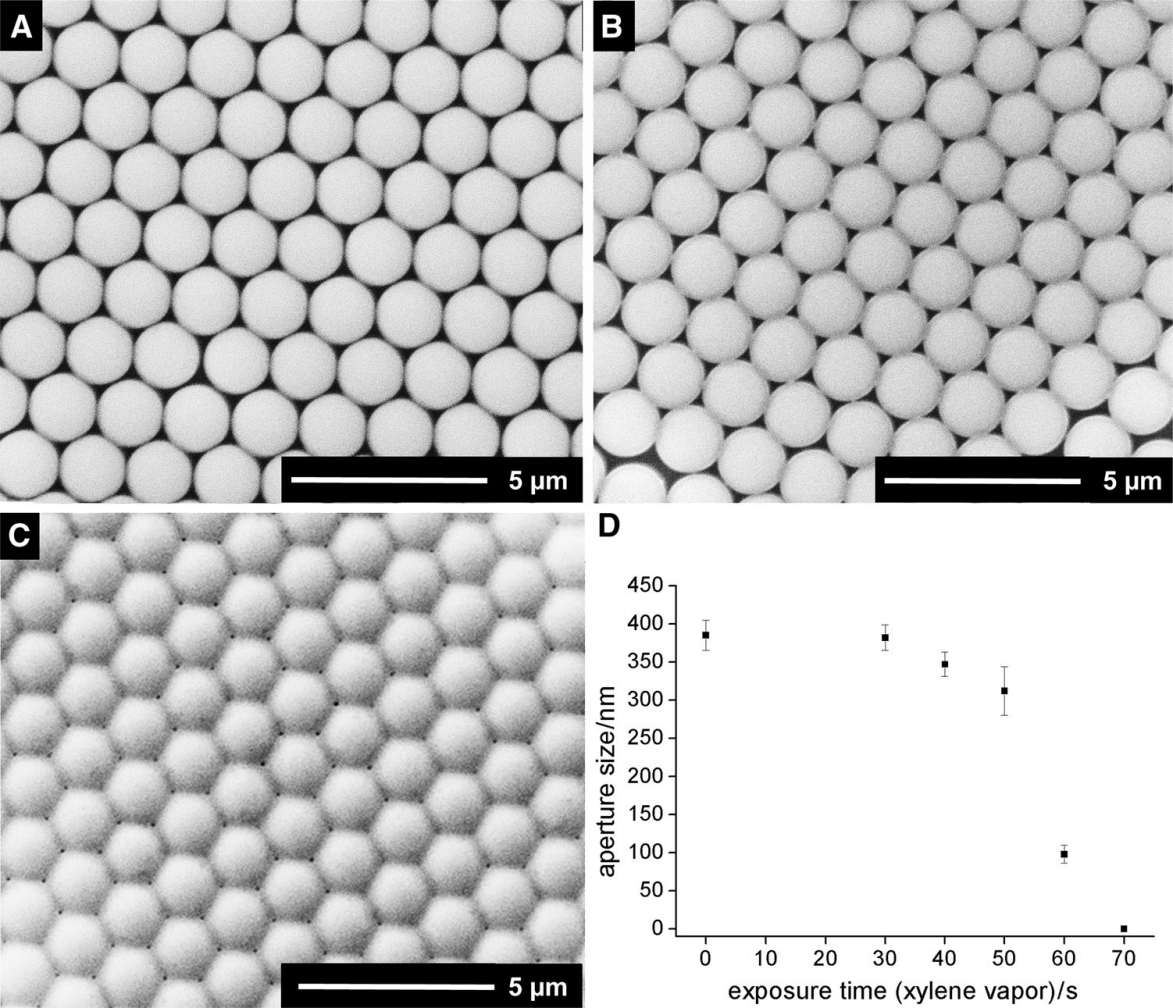
Scanning electron micrographs of PS particle films treated with dynamic vapor swelling (xylene) for **a** 40 s, **b** 50 s, **c** 60 s, and **d** aperture size vs. exposure time with xylene vapor (aperture size and standard deviation by image analysis with ImageJ for ≥100 apertures)

The same time series was conducted with xylene as solvent (Fig. 5). Exposure for 30 s did not alter the assembly, while a slight reduction in aperture size was observed after 40 s with diameters of 347 ± 16 nm (Fig. 5a). After 50 s, a crosslinked film was obtained with pore diameters of 312 ± 32 nm (Fig. 5b), similar to films treated for 40 s with toluene vapor. Thus, choosing a solvent with higher boiling point allows for a broader time window for crosslinking. Further extension of the exposure time to 60 s led to a steep decrease in pore size down to 98 ± 12 nm. As for the other tested methods, prolonged exposure times (70 s) resulted in fully closed films.

## Discussion

Static vapor swelling and dynamic vapor swelling were probed for crosslinking of floating PS particle monolayers. Static vapor swelling with divinylbenzene and subsequent UV polymerization gave access to pores in the range of 180–210 nm. Although a crosslinked film was obtained, the quality of the film is lower than static and dynamic vapor swelling methods with toluene and xylene as the dish with the floating monolayer had to be moved, in addition to the vapor swelling step, in and out of the UV chamber after the assembly and the UV polymerization, respectively. Another disadvantage of this method is that even after the UV polymerization step, residual, reactive divinylbenzene absorbed by the particles remains, leading to continued swelling and crosslinking and thus ultimately to closed films in the course of 16 h. Therefore, experiments were continued with non-reactive solvent vapors with lower boiling points, to avoid unwanted changes in pore size and allow for faster removal of absorbed solvent by evaporation.

Static vapor swelling with toluene was tested with different amount of solvents, allowing for tuning of the crosslinking time between hours and seconds. In the experiments performed with 14 cm^3^ of toluene, second-differences in exposure time make the difference between a seemingly unaffected and a completely closed film. Using a high amount of solvent in the vapor chamber (14 cm^3^) generates crosslinked films with pore diameters of 100 nm already after a short exposure time of 100 s. Thus, exposure rate and time are crucial to create monodisperse membranes with low defect density by this method.

A better control on the process for short fabrication times can be easily built in by designing a gas flow chamber (dynamic vapor swelling) allowing for controlled atmosphere for start and termination. Dynamic vapor swelling, tested with toluene and xylene vapors, allowed for the crosslinking of floating monolayers after 40 and 50 s, respectively. Pore sizes of 100–300 nm could be generated by changing the exposure time. All methods, but especially dynamic vapor swelling, were time-dependent; the transition from unaltered films to crosslinked, porous membranes and further to fully closed films occurs for dynamic vapor swelling in a time window of 10–30 s. As stated before, the time window for static vapor swelling can be tuned by the amount of applied solvent.

For all swelling methods, the area of the particle assemblies (up to 44 cm^2^) and domain sizes (up to 1.9 mm^2^) were preserved by crosslinking. However, independent of the applied method, an increase in defect density, visible in a higher number of crystal vacancies and crystal dislocations, could be observed compared to films transferred before crosslinking.

Given the identical transfer for films before and after crosslinking, the increase in defects is caused by mechanical disturbance of the assembled films during the crosslinking protocols. The defect density increased in the following order: static vapor swelling with toluene—dynamic vapor swelling—static vapor swelling with DVB coupled with UV polymerization.

## Conclusion

Highly ordered, hexagonal close-packed, crack-free, floating monolayers could be achieved by compression of PS particle films at the air/water interface using surface-active agent Triton X-100. Treatment by vapor swelling allowed for the generation of crosslinked monolayers with 100–300 nm pore diameters. The area of floating monolayers and crosslinked films can be scaled-up by merely increasing the liquid interface area and the area of the substrates used for transfer. Defect density increases with vapor annealing and transfer. Further optimization of these steps could help to prepare films with lower defect density. The best and most reproducible among the tested methods was found to be static vapor swelling with toluene. This method combines tunable crosslinking time by the amount of solvent available in the vapor chamber with a simple setup that minimizes mechanical disturbances to the assembly before completion of crosslinking.

## Experimental

All chemicals were purchased from Sigma-Aldrich and used as received unless otherwise noted. Ethanol (absolute, 99.9%) was purchased from AustrAlco. Ethanol 96% denatured was purchased from Carl Roth. Styrene was purified by passing through a column filled with inhibitor remover selective for *t*-butyl catechol. Polyvinylpyrrolidone (PVP) with an average molecular weight of 40 kDa was used as surfactant. AIBN was purified by recrystallization in methanol.

### Synthesis of polystyrene particles

A flask, charged with ethanol (102.6 g, 99.9%), was purged with nitrogen for 5 min. Styrene (15 g), AIBN (0.15 g), and PVP (1.5 g) were added under a counter stream of nitrogen. After complete dissolution, the flask was placed in a preheated oil bath (70 °C) for 24 h at a stirring speed of 100 rpm. After cooling down, the PS particles were collected by centrifugation (5000 rpm), re-suspended in ethanol (96%), and re-collected by centrifugation. This washing procedure was repeated several times. Particles were dried in vacuum.

### Cleaning of substrates

Substrates (silica, silicon) were placed in a glass tube with a solution of sodium hydroxide in MilliQ (10 wt%). The tube was placed in an ultrasonication bath for two times 15 min with changing of the substrate position after 15 min. Subsequently, the sample was washed several times with 2-propanol and MilliQ water. The sample holder was dried at 130 °C (heat gun).

### Methods

SEM: Particles and assemblies were analyzed using an FEI Inspect S50 with an ETD detector in high vacuum mode at a voltage of 20 kV. For particle analysis, 1 cm^3^ of a dispersion of PS particles in ethanol (100 µg of particle in 2 cm^3^ ethanol) was spread on a pre-cleaned silica substrate, until the surface was fully covered. Particles, assemblies, and membranes transferred to silica or silicon substrates were sputtered with a 6–7 nm-thick gold layer before imaging. Image analysis (particle size, aperture size) was performed with ImageJ 1.48v) Micrographs were processed by threshold function to generate black and white images and analyzed by Analyze Particles function.

DLS: Dispersions of particles (few micrograms/cm^3^) in ethanol were measured on a Malvern Zetasizer Nano-ZS using PMMA cuvettes. Mean values and standard deviation of number-weighted diameter were calculated from three runs.

Assembly of particles and swelling methods: Protocols for particle assembly and static and dynamic vapor swelling are given in the Supporting Information.

## Electronic supplementary material

Below is the link to the electronic supplementary material.

Supplementary material 1 (DOCX 5967 kb)
