# Supplementary material for: Crosslinking of floating colloidal monolayers
Source: Monatsh Chem. 2017 Jun 20;148(8):1539–46. doi: 10.1007/s00706-017-1997-6 (PMC5504139; doi:10.1007/s00706-017-1997-6)
Supplement: Supplementary file 1 — Supplementary material 1 (DOCX 5967 kb) [file 706_2017_1997_MOESM1_ESM.docx]

**Electronic Supporting Information**

**Crosslinking of floating colloidal monolayers**

Steffen Kurzhals,^1^ Michael Süss,^1^ Jelena Pejovic,^1,2^ Peter D. J. van Oostrum,^1^ Erik Reimhult^1^ and Ronald Zirbs^1^*

1) Department of Nanobiotechnology, University of Natural Resources and Life Sciences Vienna, Muthgasse 11, A-1190 Vienna, Austria

2) CEITEC-Central European Institute of Technology, Brno University of Technology, Purkyňova 123,

61200 Brno, Czech Republic

**Assembly at the air/water interface**


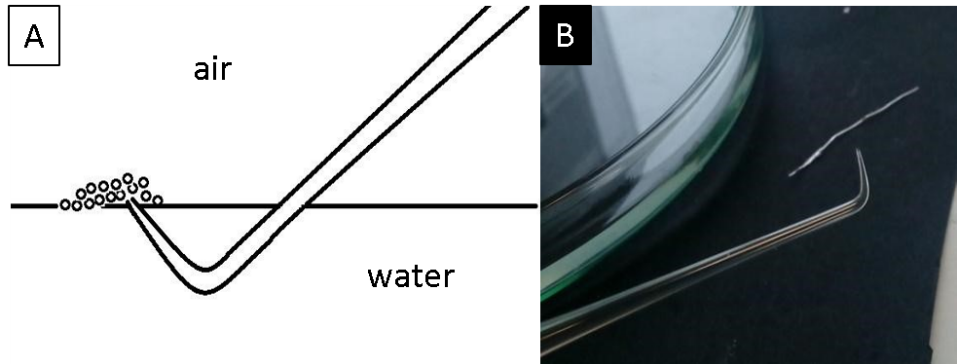


**Figure S1.** A) Spreading of PS particles at the air/water interface with pipette, B) photography of modified pipette and silver wire (length: 5 cm).


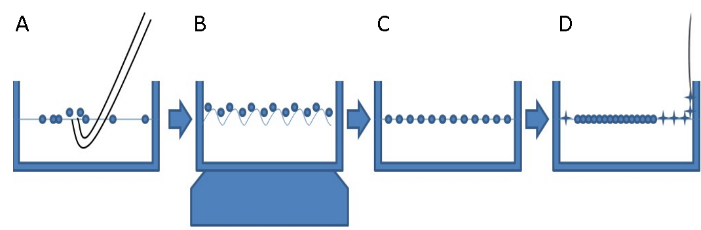


**Figure S2.** Transferring particles at the air/water interface (A), homogenizing them with tilting (B-C) and compressing with Triton X-100 (D).

The assembly procedure is adapted from Kandulski [1]. A 10 wt-% dispersion of PS particles in water/ethanol (v/v: 1/1) was used for spreading. A petri dish (diameter: 10 cm, height: 2 cm) was filled with MilliQ water (filling level: 1 cm). For a better visualization of the crystallization, the petri dish was placed on a black background. A modified pipette (Figure S1 B) filled with the PS particle dispersion was immersed into the water phase with the tip being slightly above the air/water interface, as shown in Figure S1 A and Figure S2 A. The particle dispersion was spread on the air/water interface until approximately 70 % of surface area is covered with particles. The vertical flow during the spreading was thereby kept very gently allowing a radial flow out of the particle dispersion. In the next step, the particles were evenly distributed on the air/water interface by gentle agitation for 30 min with a shaker at 45 rpm. In the last step, the evenly distributed particles were pushed together by adding small amounts of Triton X-100 (solution of 0.5 cm^3^ Triton X-100 in 2 cm^3^ ethanol) near the walls of the dish by means of a silver wire. Particle assemblies were then investigated by laser diffraction. A cleaned, pre-wetted silicon substrate was then submerged into the water phase close to the walls and moved beneath the monolayer. The monolayer was then transferred by slow lifting of the substrate.


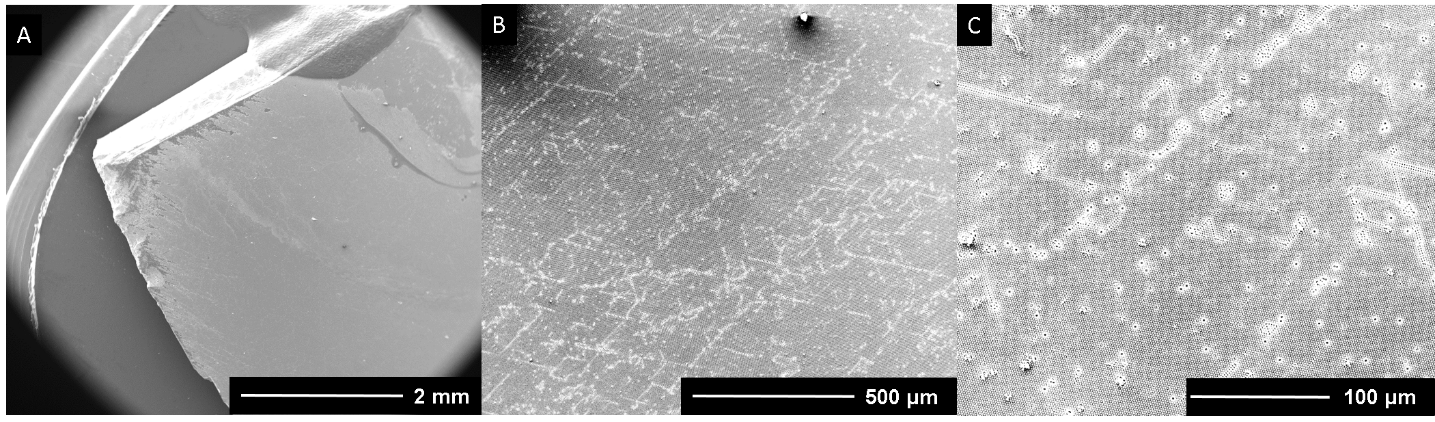


**Figure S3.** Scanning electron micrographs of PS-particle assembly at the air/water interface, gently agitated at 45 rpm for 30 min, compressed by addition of Triton X-100 and transferred to a cleaned silicon wafer.

*Crosslinking procedures*

**Static vapor swelling**


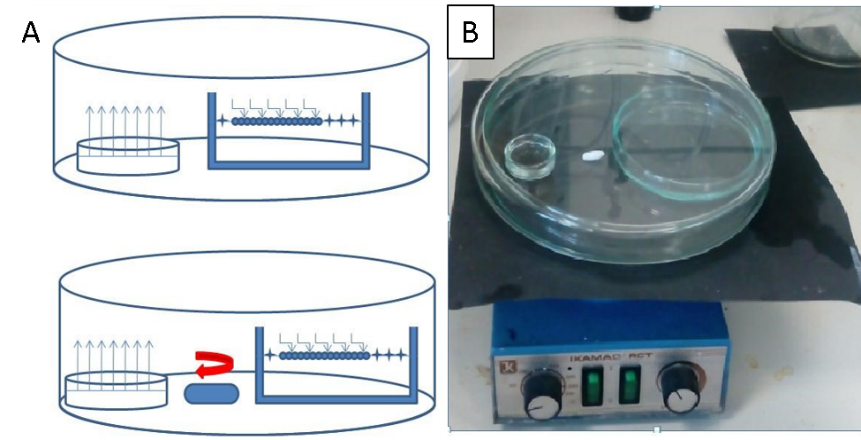


**Figure S4.** A. Scheme for static vapor swelling with or without implemented magnetic stir bar, B) Experimental setup of static vapor swelling.

**Static vapor swelling for covalent crosslinking**

A vapor chamber was constructed from a glass petri dish (diameter: 20 cm, height: 3 cm, volume: 942.5 cm^3^) and a fitting lid. A monolayer of PS-particles (assembled at the air/water interface) in a petri dish (diameter: 10 cm) was placed into the vapor chamber together with a small dish (diameter: 2.5 cm) filled with 0.5 cm^3^ DVB. Treatment with DVB vapors was performed under stirring with a magnetic stir bar (200 rpm). The dish with the swollen monolayer was then taken out of the vapor chamber, placed in a UV chamber (UV Stratalinker 1800, 4x 8 W bulbs, 3000 µW cm^-2^, 254 nm) and irradiated for 15 min. The crosslinked membrane was then transferred to a cleaned silica wafer.

**Table S1.** Parameters for Static vapor swelling/UV-Polymerization.

| entry | solvent | solvent amount  [cm^3^] | Supply with solvent | time | crosslinking /  pores |
| --- | --- | --- | --- | --- | --- |
| 1 | DVB | 0.5 | dish | 5 min | +/open |
| 2 | DVB | 0.5 | dish | 10 min | +/open |
| 3 | DVB | 0.5 | dish | 20 min | +/closed |

**
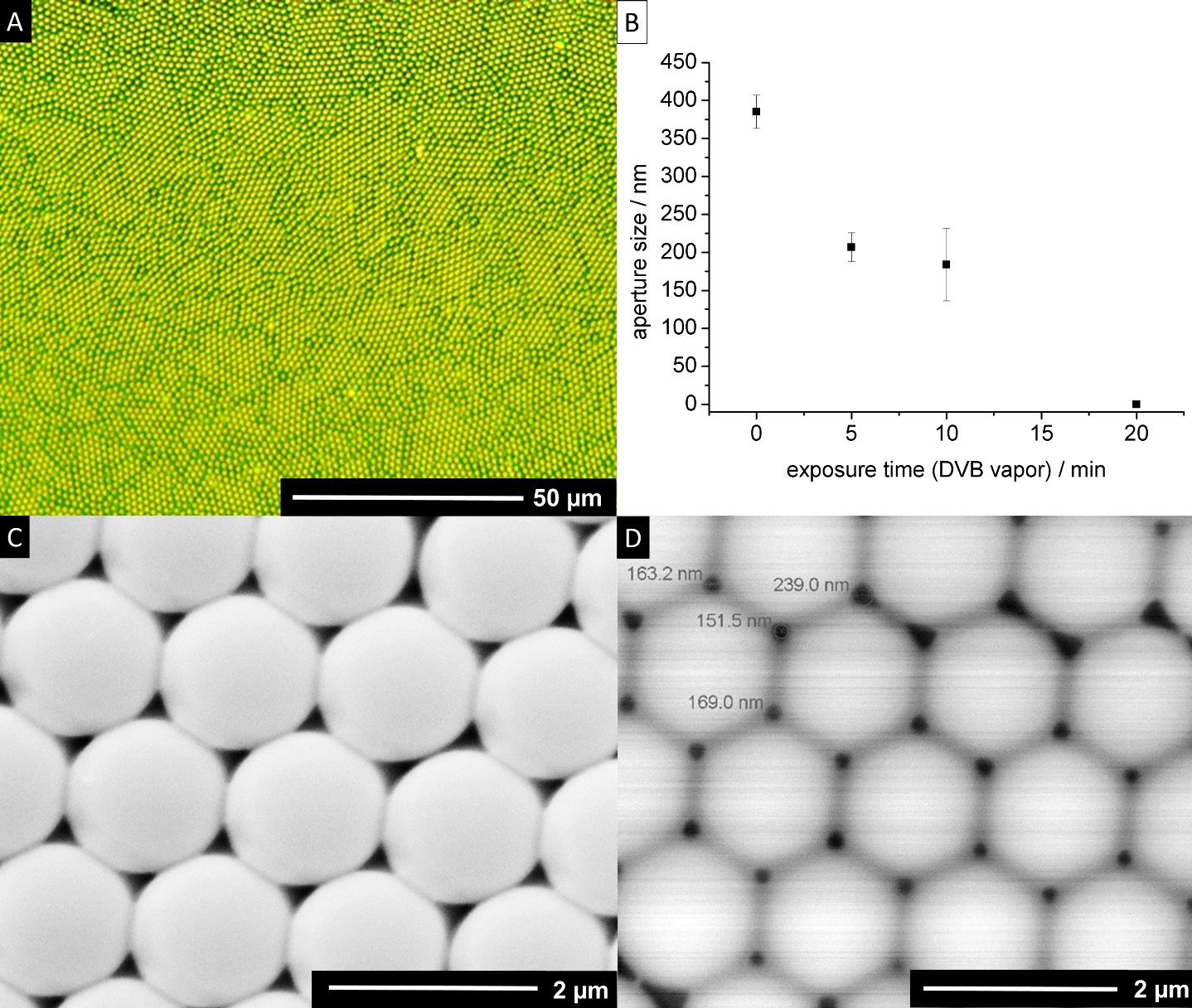
**

**Figure S5.** Dark field light micrograph of spreaded PS-particles at the air/water interface, B) Aperture size *vs.* exposure time with DVB vapor and subsequent UV-polymerization for 15 min, C-D) Scanning electron micrographs of crosslinked PS-particle assembly by DVB exposure and UV-polymerization (15 min irradiation), C) 5 min DVB exposure, D) 10 min DVB exposure.

**Static vapor swelling for physical crosslinking**

A vapor chamber was constructed from a glass petri dish (diameter: 20 cm, height: 3 cm, volume: 942.5 cm^3^) and a fitting lid. A monolayer of PS-particles (assembled at the air/water interface) in a petri dish (diameter: 10 cm, height: 2 cm) was placed into the vapor chamber. For vapor swelling either glass petri dishes (2.5 cm diameter) filled each with 0.5 cm^3^ toluene were placed in the chamber or the residual area of the chamber was covered with toluene (14 cm^3^). Experiments were performed with and without magnetic stir bar (Table S2).

**Table S2.** Parameters for crosslinking by static vapor swelling.

| entry | solvent | solvent amount  [cm^3^] |  | Supply with solvent | time | stirrer/speed | crosslinking^b^ /  pores |
| --- | --- | --- | --- | --- | --- | --- | --- |
| 1 | toluene | 0.5 |  | 1 dish^a^ | 24 h | no | unaltered |
| 2 | toluene | 1 |  | 2 dishes | 24 h | no | unaltered |
| 3 | toluene | 1.5 |  | 3 dishes | 24 h | no | unaltered |
| 4 | toluene | 2 |  | 4 dishes | 24 h | no | unaltered |
| 5 | toluene | 2.5 |  | 5 dishes | 24 h | no | +/open |
| 6 | toluene | 14 |  | full area | 90 s | no | unaltered |
| 7 | toluene | 14 |  | full area | 100 s | no | +/open |
| 8 | toluene | 14 |  | full area | 120 s | no | +/closed |
| 9 | toluene | 14 |  | full area | 95 s | yes/200 rpm | +/closed |

^a^dish with diameter of 2.5 cm, ^b^(+) crosslinking occurred.

**
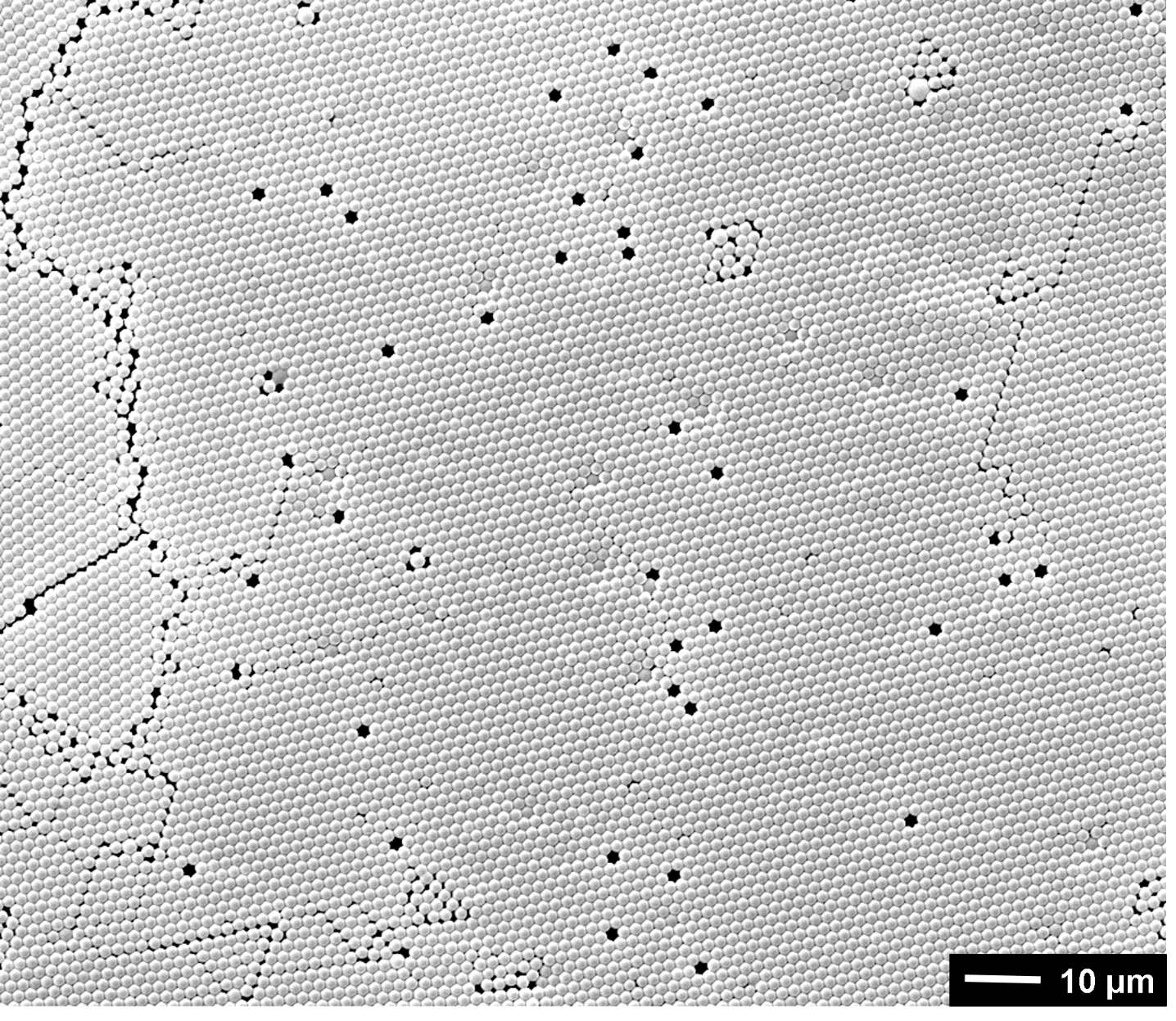
**

**Figure S6.** Scanning electron micrographs of crosslinked PS-particle assembly (Table S2, entry 7).


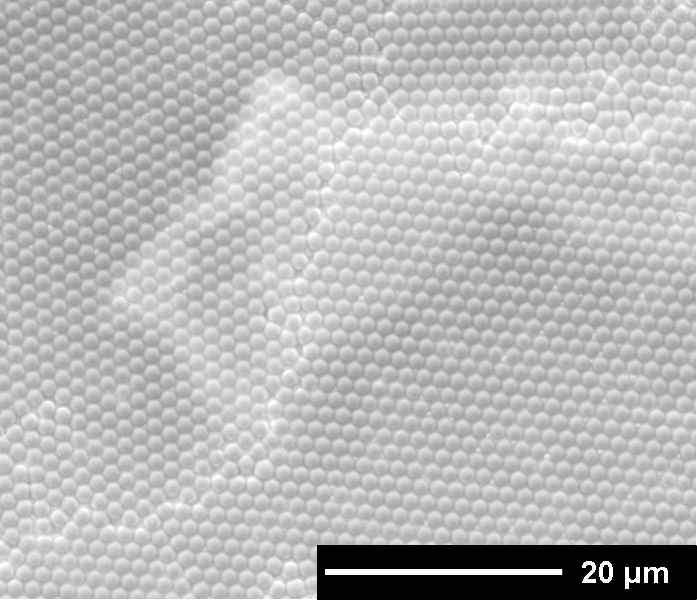


**Figure S7.** Scanning electron micrographs of crosslinked PS-particle assembly (Table S2, entry 8).

**Dynamic vapor swelling**


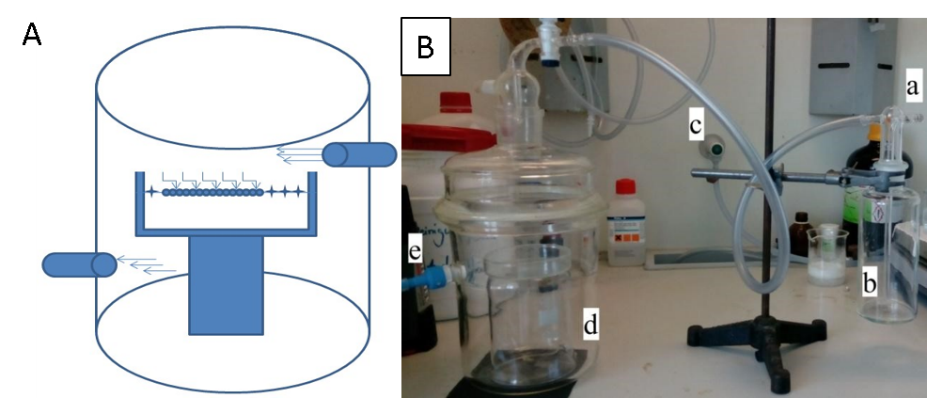


**Figure S8.** A) Scheme of dynamic vapor swelling, B) Experimental setup, a: connection to air, b: washing bottle with solvent, c: connection tube to vapor chamber, d: vapor chamber, e: connection to vacuum.

**Table S3.** Parameters for crosslinking by static vapor swelling process

| entry | solvent | solvent amount  [cm^3^] |  | time  [s] | crosslinking^a^ /  pores |
| --- | --- | --- | --- | --- | --- |
| 1 | toluene | 14 |  | 120 | unaltered |
| 2 | toluene | 100 |  | 30 | unaltered |
| 3 | toluene | 100 |  | 40 | +/open |
| 4 | toluene | 100 |  | 60 | +/closed |
| 5 | xylene | 100 |  | 30 | unaltered |
| 6 | xylene | 100 |  | 40 | unaltered |
| 7 | xylene | 100 |  | 50 | +/open |
| 8 | xylene | 100 |  | 60 | +/closed |
| 9 | xylene | 100 |  | 70 | +/closed |

^a^(+) crosslinking occurred.

The setup for dynamic vapor swelling is depicted in Figure S8. Air is sucked through a washing bottle filled with solvent. The solvent-saturated air flows over the self-assembled monolayer for a defined amount of time (30-120 s) resulting in a swelling of the particles. Tested conditions are summarized in Table S3. For experiment (Table S3, entry 1), air is sucked over solvent, while for entries 2-9, air is sucked through solvent. The process was stopped by closing the valve on top of the vapor chamber, resulting in immediate removal of the solvent-saturated atmosphere from the vapor chamber.

**References**

1. Kandulski W (2007) Shadow Nanosphere Lithography. PhD-Thesis, Rheinische Friedrich-Wilhelms-Universität Bonn, Bonn
